# Cyclone avoidance behaviour by foraging seabirds

**DOI:** 10.1038/s41598-019-41481-x

**Published:** 2019-04-01

**Authors:** Henri Weimerskirch, Aurélien Prudor

**Affiliations:** Centre d’Etudes Biologiques de Chizé, CNRS, 79360 Villiers en Bois, France

## Abstract

In the context of climate change, how extreme climatic events, such as cyclones, will affect the foraging abilities of marine vertebrates is still poorly known. During the course of a study on the foraging behaviour of two tropical seabirds, red-footed boobies and great frigatebirds, several cyclones have affected their breeding grounds and foraging zones, allowing us to study their response to extreme wind conditions. We examined whether adults and young naïve birds were able to predict the arrival of a cyclone and behave accordingly to reduce mortality risks and optimise foraging. We show that when a cyclone approached, juveniles and adults of the two species differed in their decisions to leave the colony for the sea. When the winds reached gale force, the juveniles of both species and adult frigatebirds remained at the colony, whereas adult boobies continued their foraging routine. The mortality of the individuals remaining on land remained limited. When encountering at-sea gale conditions, adult birds were able to avoid the centre of the low pressure systems and moved westward to bypass the route of the cyclones and circumvent the moving cyclone. Frigatebirds climb to high altitudes when close to the eye of the cyclone to bypass it at high speeds. These movements likely reduce the mortality risk at sea but can temporarily cause birds to move outside their normal range at sea or over land masses. We discuss the potential consequences of an increase in cyclonic conditions on seabird populations.

## Introduction

During their lifetimes, animals move in time and space to forage for food, find mates, and avoid adverse conditions or predation. Movement decisions are therefore a critical determinant of individual fitness and are likely a trait under strong selection^1^. Adopting optimal movement decisions to avoid adverse conditions will be fundamental in the context of climate change^2,3^. Extreme climatic events are predicted to increase in frequency under climate change^4^ and can have important negative effects on the dynamics of populations^5,6^. Tropical cyclones are extreme climatic events that are well known for their devastating effects on terrestrial and marine ecosystems^7,8^. Over the past decades, the zone of cyclone occurrence has been shown to move poleward^9^, and the strength of cyclones has increased^10^. Future climate changes are expected to be associated with an increase in the strength of these extreme climatic events^4,11^. However, our understanding of how tropical cyclones currently affect marine biodiversity, particularly pelagic species, is limited, reducing our ability to make predictions about the future effects of this climatic phenomenon on biodiversity.

The effects of cyclones on terrestrial habitats and species can be devastating^7,8^. Cyclones form and occur primarily at sea and can have profound disturbance effects on marine ecosystems by inducing bottom-up forcing^12^. Very little is known about the behavioural response of marine animals to a cyclonic event. Birds are sensitive to wind conditions during migration or when foraging at sea, and seabirds are particularly vulnerable to gales since they cannot find shelter when facing extreme wind conditions in the open sea^13^. It is well known that seabirds can be displaced outside their normal range and even well inland following major cyclones^14,15^, suggesting that they could be unable to avoid cyclones. Thus, an increase in cyclone intensity is likely to increase the mortality of seabirds and to increase the risk of extinction in the rarest tropical species^14^. In the context of future climate change and extreme climatic events, such as cyclones, it appears important to understand and predict how individuals will respond to these extreme events.

Since tropical seabirds have evolved with regular encounters with cyclones during their long lifespans, it is likely that specific behaviours have been under selection to reduce the potential negative effects of cyclones when foraging at sea^16^. Long-range movements have to be constantly adjusted to local conditions, particularly to minimise energy expenditures^17,18^ and avoid mortality. Therefore, birds should modify their migratory or foraging behaviour accordingly to avoid deleterious effects^19^. It has been suggested that birds may forecast the arrival of cyclones and avoid them through their capacity to forecast cyclone arrival based on associated meteorological conditions (wind, barometric pressure) or by hearing infrasound^20^. Indeed, extreme climatic events, such as cyclones, produce infrasound that can be physically detected more than 1000 km from the phenomenon^21^. Birds could be able to detect powerful thunderstorms hundreds to thousands of km away through infrasound detection^22^. However, the ability to forecast the arrival of a cyclone may simply be through environmental cues associated with the cyclone, such as wind strength and direction, atmospheric pressure or cloud cover^23,24^.

The aim of this study was to examine the behavioural responses of two seabird species to the approach and passage of a cyclone. We studied the foraging behaviour of great frigatebirds (*Fregata minor*) and red-footed boobies (*Sula sula*) when cyclones crossed the breeding and foraging grounds of these two species. We were specifically interested in whether adults and naïve juveniles make different decisions to leave the colony and forage at sea and their at-sea foraging behaviour when the cyclone approaches and thus whether some forecasting and adaptation of their movements occur. The comparison between the two species is of interest because they have very different morphologies and flight strategies. Frigatebirds have the lowest wing loading among birds, with a unique capacity for soaring flight^25^, are unable to land on the sea surface or dive even though they feed exclusively at sea, and can stay aloft for months during long foraging movements^26^. Conversely, red-footed boobies are strong flyers with long, narrow wings that are used to flap-glide fly under windy conditions at low costs^27^, are able to sit on the water when foraging, and plunge dive frequently^28^. Thus, in view of these differences, we predict that these two species may respond differently to cyclonic conditions. The results should help in the understanding of the susceptibility of these two groups of seabirds to future climate change.

## Results

**Figure 1:**
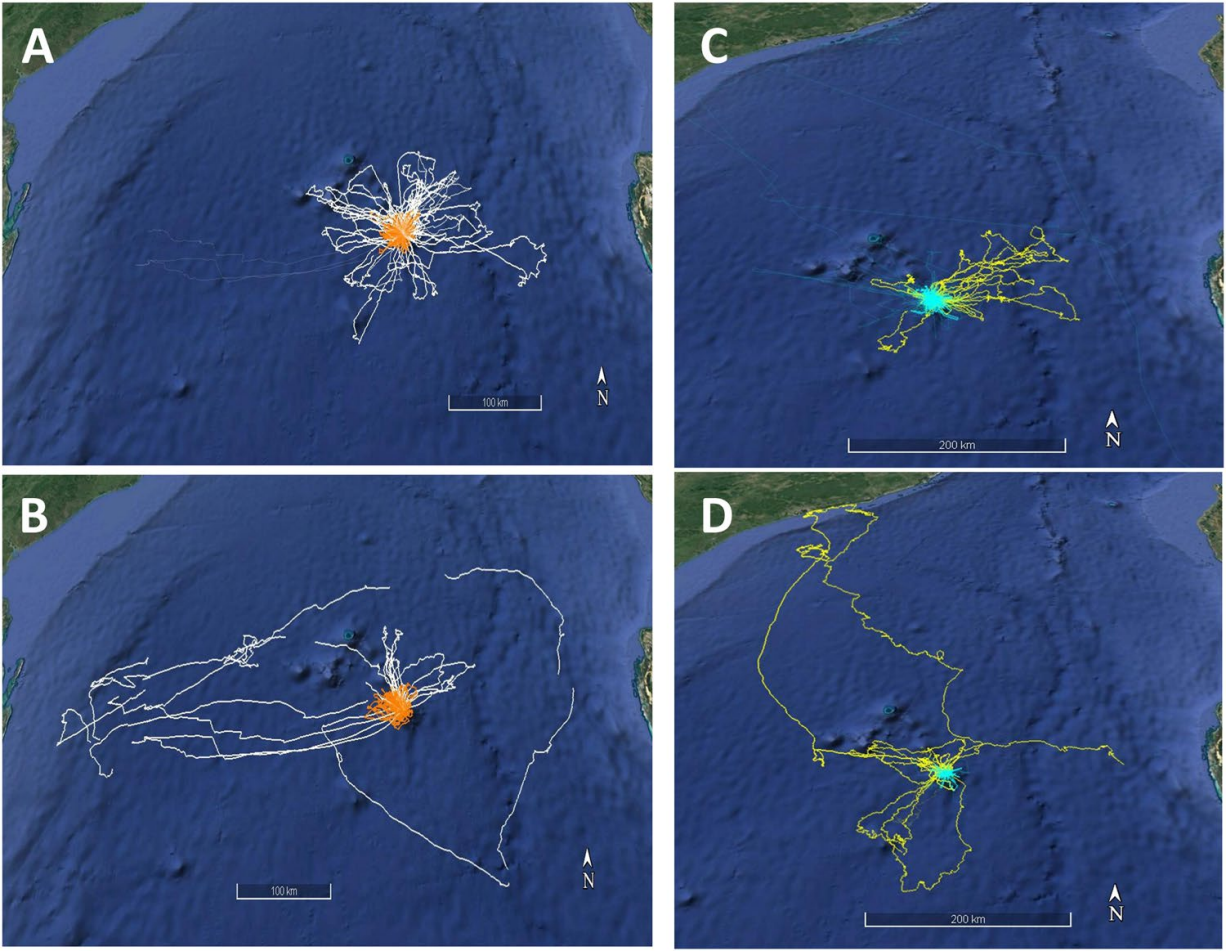
GPS tracks of (**A**) Foraging trips of adult (white) and juvenile (orange) red-footed boobies before the arrival of cyclone Guito (14-18/2/2015) and (**B**) during cyclone Guito (19-21/2/2015). (**C**) Foraging trips of adult (yellow) and juvenile (blue) great frigatebirds before and (**D**) during cyclone Guito. Maps created from Google Earth (© 2018 Google; Data SIO, NOAA, US Navy, NGA, GEBCO; Image Landsat/Copernicus).

**Figure 2:**
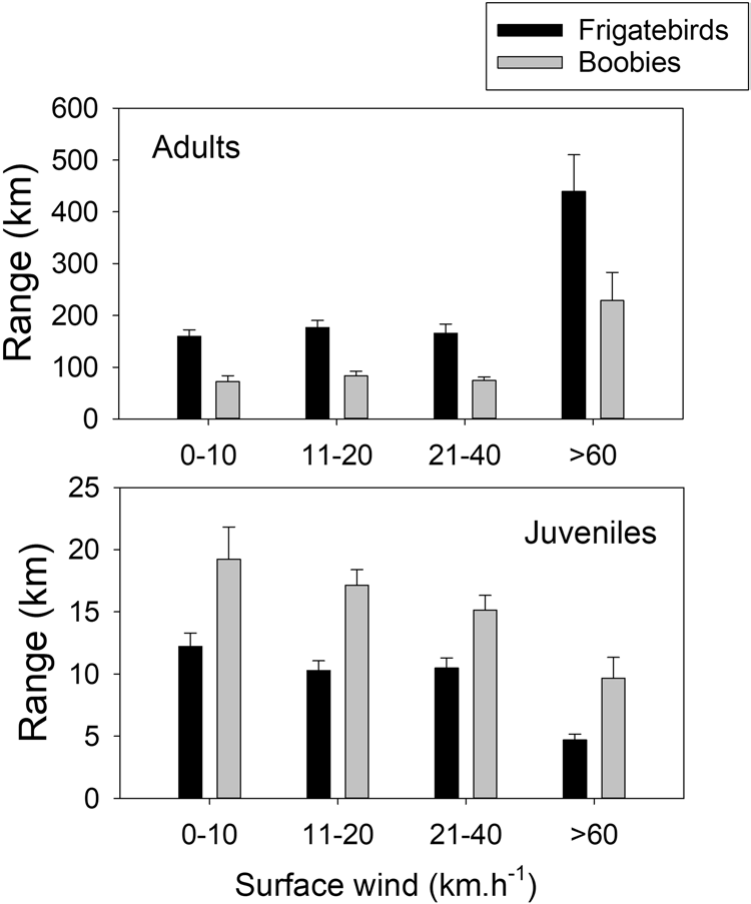
Foraging range (mean ± one S.E.) of adult and juvenile boobies (grey) and frigatebirds (black) under increasing wind conditions.

The average surface winds recorded on Europa varied between 0 and 40 km.h^−1^. Average winds stronger than 60 km.h^−1^ were associated with an intense tropical depression or cyclone (Supplementary information, Fig. S1). Before the arrival of the cyclone in the Europa area, with surface winds varying between 0 and 40 km.h^−1^, adult boobies performed daily trips at sea lasting 9.8 ± 3.8 h and returned every night to the colony, and adult frigatebirds performed trips lasting 17.8 ± 30.0 h (range 1–7 days) and stayed aloft constantly. Juveniles of both species conducted shorter daily foraging trips than adults in the vicinity of the island (boobies 5.6 ± 4.5 h, frigatebirds 3.2 ± 4.4 h) and did not travel as far as adults (Figs 1 and 2).

**Figure 3:**
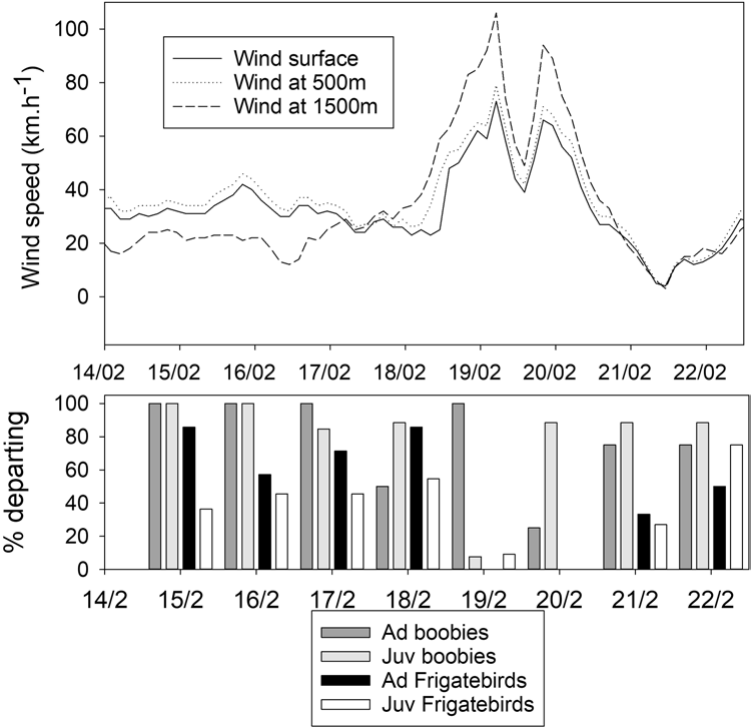
Surface wind and winds at 500 and 1500 m on Europa (values from https://earth.nullschool.net) and percentages of birds present on the colony and departing every day from the island.

Among the adults, the foraging ranges were larger for frigatebirds than for boobies (F_1,12_ = 5.2, P = 0.036), whereas for juveniles, the ranges were smaller for frigatebirds than for boobies (F_1,29_ = 11.6, P = 0.002) (Fig. 2). The majority of the tracked birds present on the island left the colony every day, mostly in the morning (Fig. 3). When the cyclone approached and the eye was 250 km from the island on the night of the 19^th^ of February and the wind increased to gale conditions (average ground speed > 60 km.h^−1^), adult frigatebirds stopped leaving the island for their foraging trips at sea and remained on the island, whereas most adult boobies continued to leave to go out to sea (Fig. 3). Most juvenile boobies and frigatebirds remained at the colony when the cyclone approached (Fig. 3, juveniles). The few juveniles that left the island when the cyclone was only 200 km away from Europa left very early in the morning and made very short trips before returning to the island.

**Figure 4:**
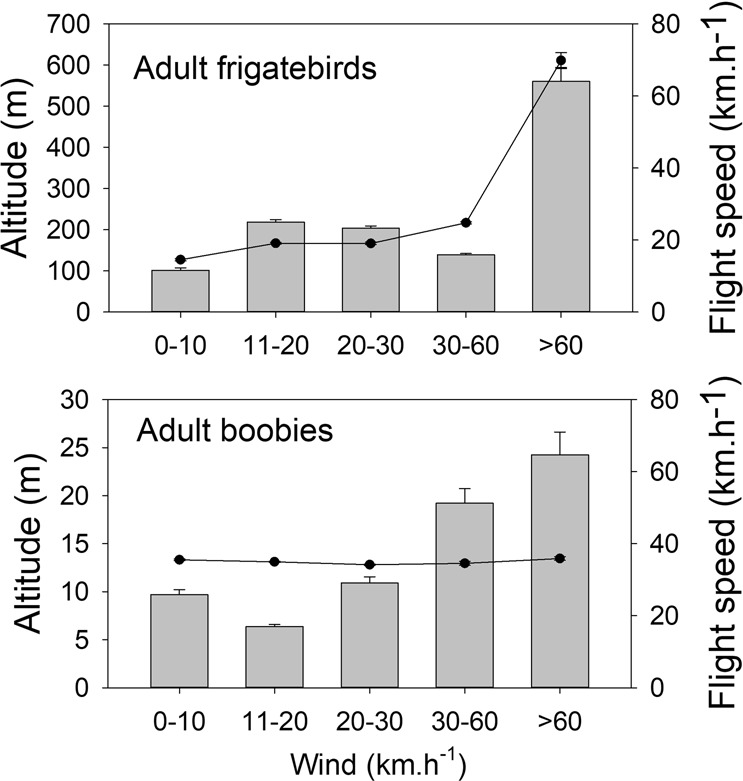
Mean (± one S.E.) altitudes and flight speeds of adult boobies and frigatebirds associated with the different surface wind speeds encountered at each GPS location.

**Figure 5:**
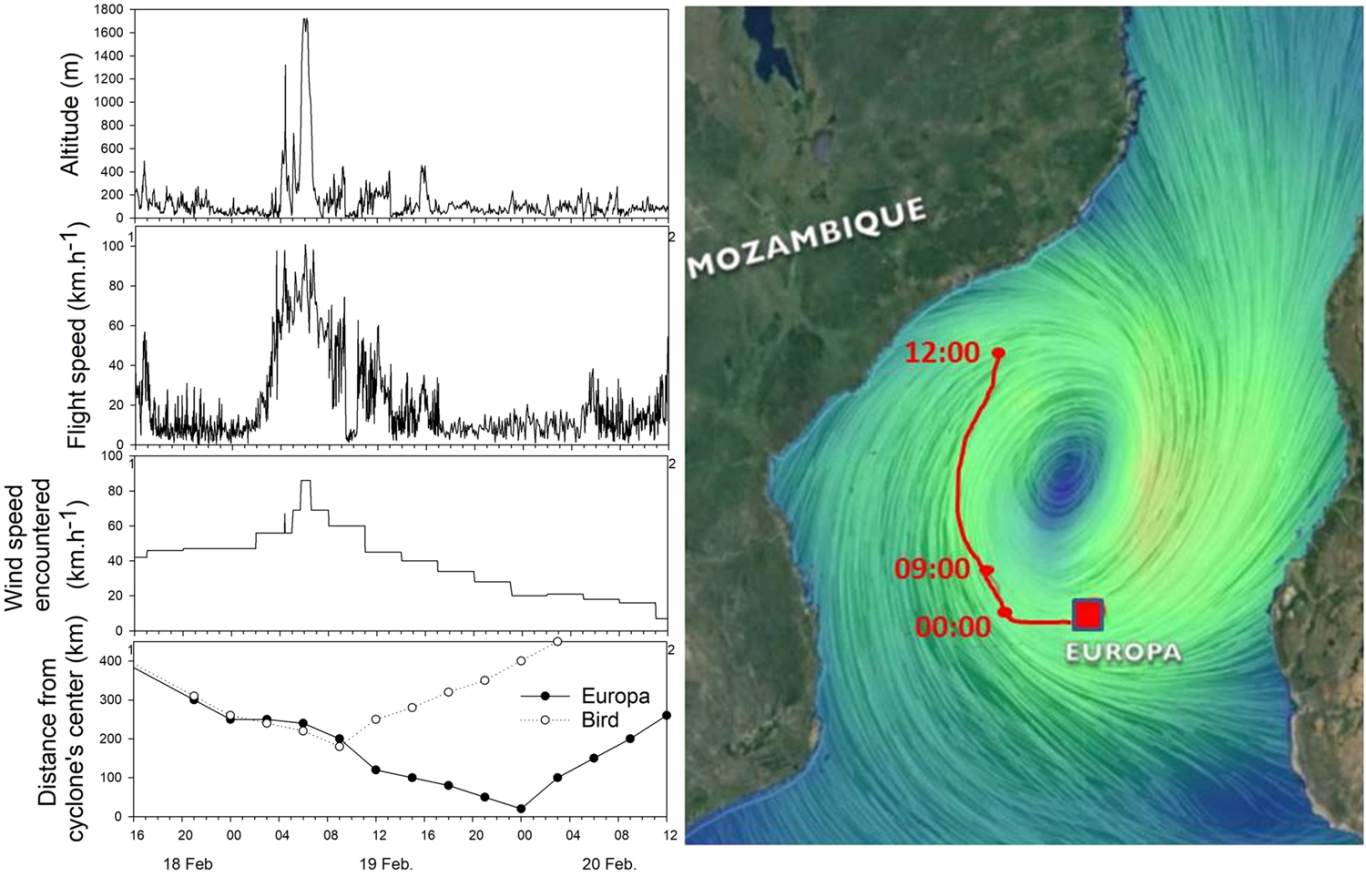
Left: Flight parameters of and wind speed encountered by a frigatebird by passing-cyclone Guito and the corresponding movement on 19/02 between 00:00 and 12:00 when the bird circumvented the cyclone (created from SigmaPlot v13). Right: The southward moving cyclone showed a typical clock-wise movement around the eye (image redrawn from https://earth.nullschool.net, with trajectory from Google Earth, (© 2018 Google; Data SIO, NOAA, US Navy, NGA, GEBCO; Image Landsat/Copernicus)).

Adult boobies leaving for the sea during gale winds moved westward at a much longer range than during the previous days (F_1,5_ = 14.1, P = 0.023) and returned to the colony after 3–7 days (Fig. 1). Frigatebirds at sea when the cyclone approached did not return to Europa and therefore made longer trips in terms of range (Figs 1 and 2) and duration. In adults, the distances were longer under cyclonic conditions in both species compared to those under normal wind conditions (Fig. 2, F_3,13_ = 10.3, P = 0.035), whereas for the few juveniles that were at sea during the cyclonic conditions, the ranges were shorter (Fig. 2; F_3,30_ = 2.8, P = 0.048). Adult frigatebirds flew at higher altitudes on average with gale winds and had a higher flight speed (F_4,12_ = 15, P = 0.052 and F_4,12_ = 15, P < 0.01, respectively) (Fig. 4). One adult frigatebird that was at sea before the arrival of the cyclone circumvented the cyclone westward. When being the closest to the eye of the cyclone (180 km) on its overall trajectory, the bird climbed to a high altitude (up to 1600m) and moved quickly northward (Fig. 5).

Boobies generally fly close to the surface, but during the cyclonic event, the three adults escaping the cyclone regularly climbed to high altitudes, reaching 1100 m for one bird, before returning close to the sea surface. This behaviour was not related to gale winds in particular, but there was a tendency for birds to fly at a high altitude with strong winds (F_1,5_ = 2.8, P = 0.065, Fig. 4). Flight speed was not affected by wind speed (F_1,5_ = 0.3, P = 0.87).

**Figure 6:**
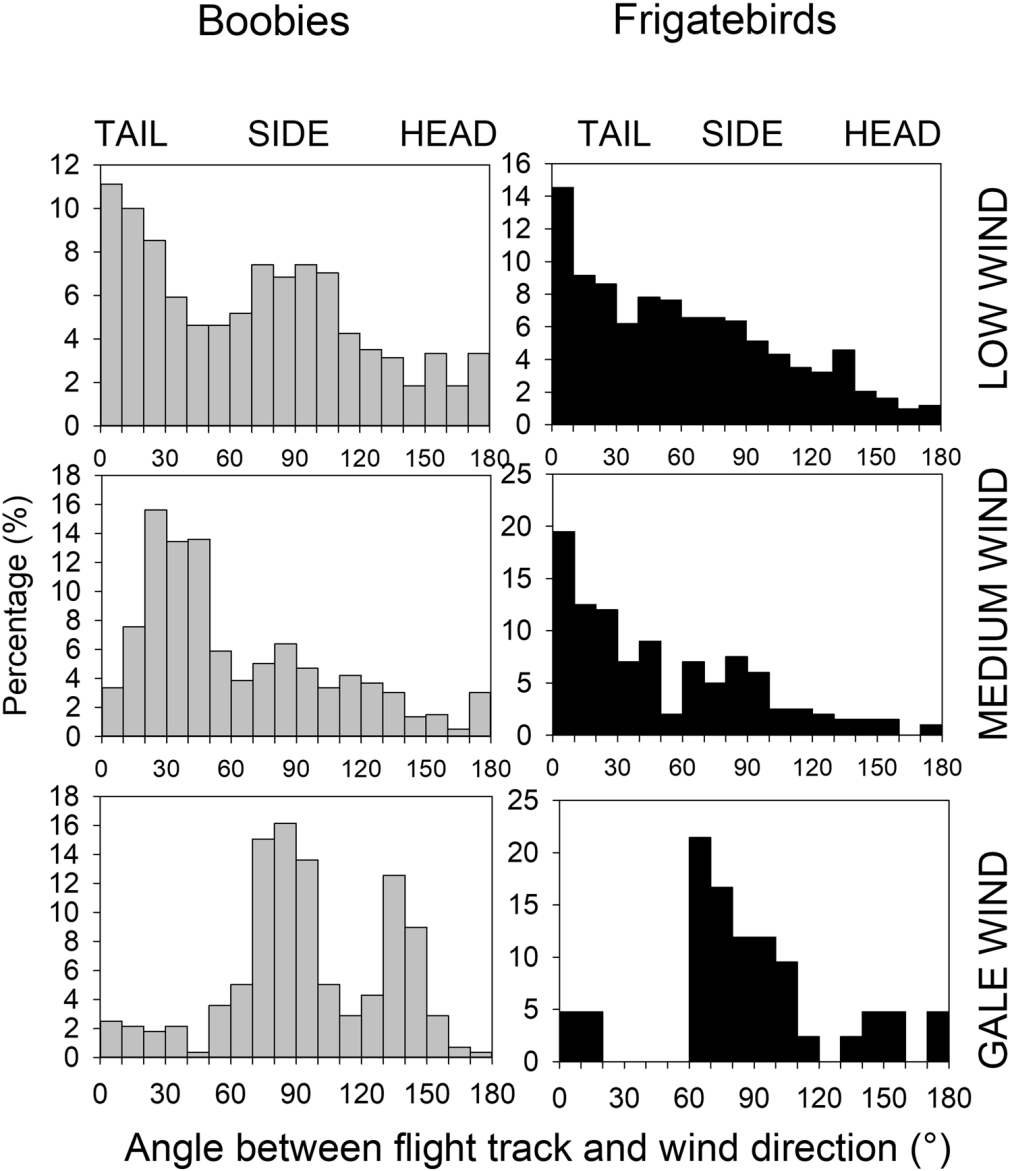
Distribution of flight tracks with regard to wind direction encountered for each GPS location in boobies and frigatebirds under various wind conditions (low: 0–20 km.h^−1^; medium: 21–60 km.h^−1^; gale: >60 km.h^−1^).

When flying under gale conditions, birds of both species use wind differently compared to when they travel with weaker winds. When flying in the presence of weak winds, both species preferentially fly with a tail wind and to a lower extent with a side wind when the wind force increases (Fig. 6). Under gale wind conditions, both species use only side winds. Frigatebirds, which are continuously climbing by soaring and descending by gliding at an average altitude of 100–200 m, climb to higher altitudes during gale winds and stay at a high altitude (Fig. 5), where they attain very high travel speeds (Fig. 6).

The cyclone partly destroyed the forest where the boobies and frigatebirds nest. Of the 31 juvenile and 4 adult boobies fitted with loggers and present on the island during the passage of the cyclone, two juveniles were killed (5%). For the frigatebirds, of the 8 adults and 14 juveniles equipped with loggers and present on the island during the cyclone, none were killed. However, a few non-equipped juvenile frigatebirds in the colony were found on the ground that had been killed by falling trees or branches.

**Figure 7:**
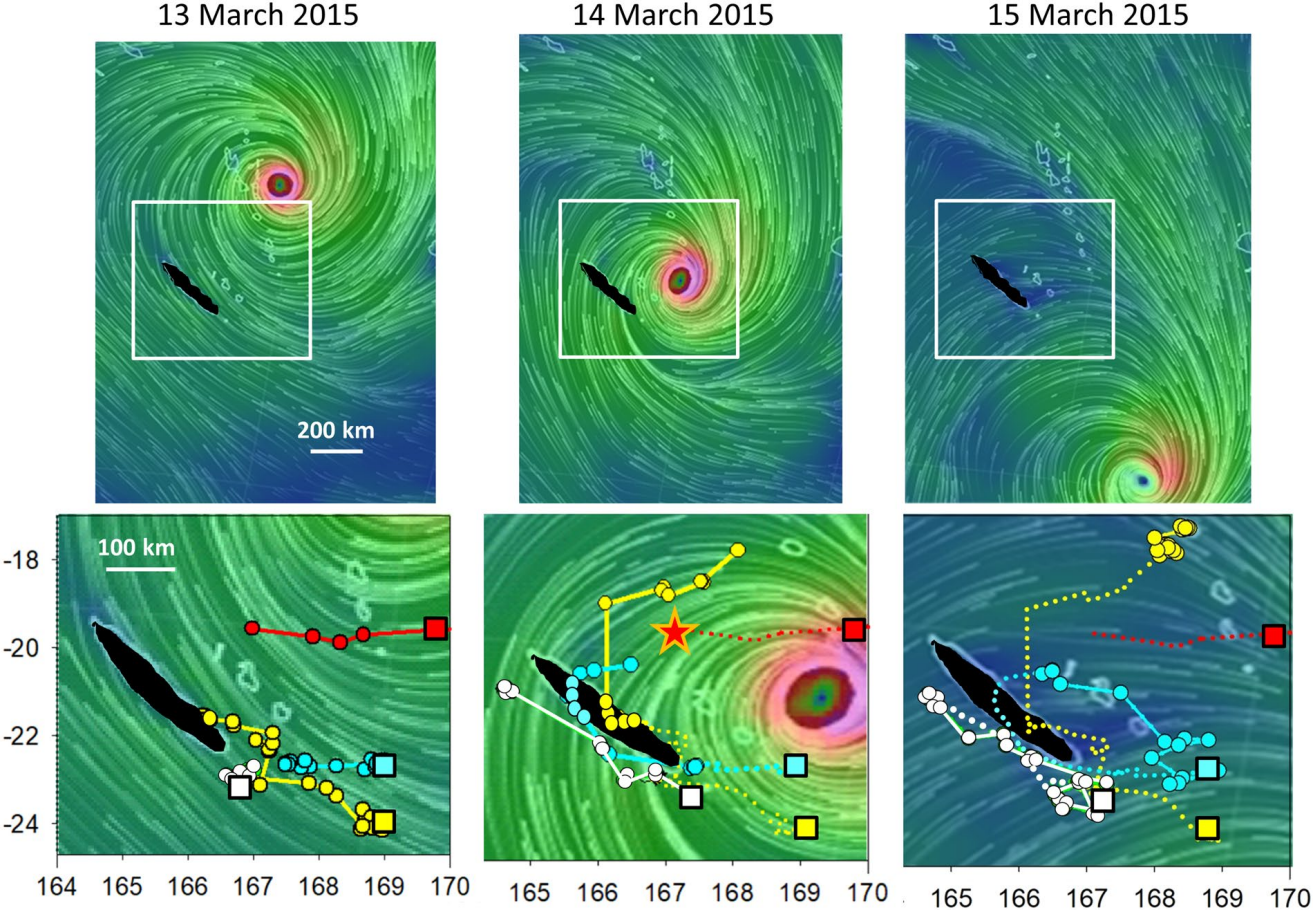
Top: southward trajectory of category 5 cyclone Pam between 13 and 15 March (images created from SigmaPlot v13, redrawn from https://earth.nullschool.net), with the zone enlarged in the lower panel indicated by the white square. Bottom movement of 4 frigatebirds, with squares representing the first locations, the dots and lines indicating the movement during the day considered, and the dotted line indicating the trajectory on the previous days. On 13 March, when the cyclone approached all 4 birds moved westward; on 14 March, when the eye was at its closest from the starting point, birds had moved 400 km westward and then circumvented the border of the cyclone, but for the closest bird (red dots and lines) to the eye the transmitter stopped – suggesting that the bird was killed – (red star). On the 15^th^, two birds moved back to their starting points, one moved northward and stopped in Vanuatu 600 km from the starting point.

In New Caledonia, when cyclone Pam approached from the island with southward movement, strong easterly winds were blowing at the front of the cyclone. Four individuals were foraging from neighbouring islands at short ranges (10–40 km), and when the cyclone eye was 470 km from the closest bird and 800 km from the 3 others on the surrounding islands of New Caledonia, with easterly winds at the front of the cyclone, they all moved west and then to the north, with 2 birds crossing over the New Caledonian mainland. The birds that were the closest to the eye (250 km at the closest to the cyclone eye, with wind > 100 km/h) died, two birds returned to their original foraging zone after the cyclone continued its route towards the south, and one bird was displaced to Vanuatu, 600 km to the north (Fig. 7).

## Discussion

To be able to make predictions on the impact of increasing extreme climatic events on seabird populations under climate change, it is critical to understand the ability of foraging seabirds to reduce the risk of deleterious effects. This study is the first to document how foraging seabirds respond to the presence of cyclones. It shows that two species with contrasting flight capacities have different responses to the arrival of a cyclone and that naïve young individuals react differently from adults.

Cyclones are frequent in the Mozambique channel and around New Caledonia; one or several cyclones occur every year, and one hit Europa Island or New Caledonia almost every year during the past 5 years, suggesting that seabirds from these island regularly experience cyclonic conditions and have likely evolved behavioural strategies to avoid detrimental effects^16^. The foraging decisions when the cyclone approached were different and appear to be linked to species morphology and the associated ability to fly without risk in gale winds and to the age and breeding constraints of the birds.

Frigatebirds, with their long and wide wings adapted for soaring flight, inability to sit on the water, and fragile body structure, are likely the most susceptible to gale winds. The adult frigatebirds reacted to the approach of the cyclone when the winds reached gale force. When at sea or non-breeding on small islets, they are able to avoid the centre of the cyclone eye, where the winds are the strongest, by moving westward at the edge of the cyclone and then circumventing the cyclone using a circular movement around the progressing cyclone. The New Caledonian bird movements were remarkable because four birds evacuated the roosting sites one day before the cyclone reached the island, with similar and simultaneous westward movements. Thus, frigatebirds have developed a strategy that allows them to circumvent cyclones, and, when they are the closest, to continuously stay at a high altitude when escaping the cyclone centre, probably to avoid more turbulent conditions closer to surface^29^. By using side winds during gale conditions instead of tail winds, they are pushed away from the advancing cyclone, which has a clockwise movement, thus allowing them to bypass the cyclone instead of being pushed in front of the cyclone. This strategy of moving on the western side of the cyclone and birds drifting clockwise around the cyclone and avoiding the eye is suggested by theoretical modelling work^30^. Under these conditions of extreme winds, the birds likely cannot entirely control their route and can be pushed into zones outside their normal range or over land masses that they never frequent otherwise^15,31^. Compensation for drift is likely not possible in such strong winds, but drifting on the outer western edge of the cyclone should generally be a safe strategy. Mortality likely still occurs, as indicated by the death of the bird that was the closest to the eye of the very intense cyclone Pam. Adult boobies are more strongly built birds compared to frigatebirds and use flapping-glide flight; they still continue to leave the breeding site for foraging trips, and, similarly to frigatebirds, use an escape flight strategy of flying with side winds at high speed to move away from the cyclone eye by moving westward and return to the island after the cyclone continues its route.

Juvenile birds of both species, which have small foraging ranges and are in their transition phase before being left alone by their parents, appear to be able to predict the arrival of a cyclone and stay ashore as it approaches. However, staying at the colony has a cost, as indicated by the mortality due to falling trees.

Our study suggests that the arrival of gale winds at the front of the cyclone is sufficient to allow frigatebirds to determine the proximity of cyclonic conditions and either stay ashore if they are in the breeding colony or circumvent the cyclone if they are at sea or non-breeding. They made these decisions when the eye of the cyclone was 250 km away from the colony with a medium intensity cyclone and 600 km away from non-breeding sites with a very strong cyclone. This result suggests that the perception of wind conditions preceding the cyclone may be the signal that allows them to forecast the risks of foraging in the coming hours. Birds may forecast cyclone arrival based on associated meteorological conditions (wind, barometric pressure) or infrasound. The ability of birds to detect infrasound^32^ has led to the hypothesis that birds may be able to forecast the arrival of a storm or cyclone and make a decision to avoid it^20^. Since infrasound can be perceived by sensors up to 1000 km away^21^, birds may make decisions much ahead of a cyclone^19^. In our study, seabirds appear to be able to circumvent cyclones but use avoidance behaviours only when a cyclone is relatively close and likely when they perceive a wind strength and direction that is indicative of the movement of the cyclone and its heading. Indeed, in the Southern Hemisphere, cyclones have a general southward clockwise movement, with wind turning around the eye, and increase in strength when approaching the eye. Thus, a bird to the south of the approaching cyclone would encounter easterly winds (Fig. 3). This information may be used by seabirds to promote escape and circumvent the cyclone, precluding them from being on the route of the cyclone by adopting a strategy of flying with side winds when the winds attain gale force, and they thus do not show the typical foraging movement from the island, as our results indicate. Songbirds are similarly able to circumvent tornadic storms during breeding^19^.

Our results indicate that adult frigatebirds and boobies are able to predict the imminent arrival of cyclones and to bypass them, suggesting that cyclones may not result in the high mortality of adults, as expected for long-lived animals. On land, cyclones have devastating effects on seabird nests and colonies and thus can strongly affect breeding success^33,34^. In tropical seabirds, as in birds in general, the timing of breeding has likely been selected to maximise fitness^35^. Boobies and frigatebirds breed in trees, and nesting is susceptible to strong winds^33^. The incubation and brooding of young chicks occur before the cyclonic season^36^, reducing risks of breeding failure at this critical stage. Our results on the behavioural response of these two tropical seabirds are in agreement with the results of the few demographic studies available on tropical species. Cyclone activity seems to not affect the annual mortality of adult tropical petrels^6^ but may affect juvenile survival when occurring in the breeding grounds, as our results also suggest. Similarly, the survival of young boobies is more affected by the local climate conditions than that of adults^37^, and the survival of adult boobies and tropicbirds was not affected by extreme climatic events^38,39^. The foraging and dispersive ranges of frigatebirds and boobies are in zones with the frequent occurrence of cyclones, and these species have evolved adaptive strategies that reduce the risks associated with the potential for experiencing these extreme events. In particular, frigatebirds encounter several atmospheric challenges during their movements at sea in addition to cyclones, such as low temperatures, low air density and oxygen levels during high climbs, and the unpredictable distribution of cumulus clouds at the small scale^26^. Climate models for the tropical ocean forecast an increase in the intensity of tropical storms and of convections around the Equator, where doldrums and strong convections occur^40,41^. With the increasing temperatures and variability related to large-scale events^42^, the more variable atmospheric conditions in the future may become very challenging for a species that already seems to regularly encounter extreme conditions during the movements over its lifetime. Conversely, boobies may be less susceptible to the stronger cyclones in the future compared to frigatebirds because of their ability to sit on the water and their stronger morphology. In long-lived animals, in which changes in behaviour can occur faster than evolution, different age classes may vary in their adaptation to climate change^43^. Juvenile birds that stay on the island when a cyclone approaches are  probably more vulnerable to mortality on land since they do not use an escape behaviour, likely because they have not yet attained proper flight and foraging skills like adults. However, being at sea during cyclonic conditions may be even more deleterious for these naïve young animals, and selection appears to have favoured the best of two poor scenarios.

In conclusion, our study shows that two tropical species have evolved movement behaviours that reduce the risk of mortality at sea for adults, the most vulnerable age class in long-lived animals. Young naïve birds of both species fledge during the cyclonic season but reduce the risk of encountering a cyclone at sea by staying ashore. However, some species may have important traditional foraging zones in the path of cyclone trajectories and could be threatened by increasing cyclone frequency^14^. Thus, future studies predicting the impact of climate change and extreme climatic events should take into account that future susceptibility to climate change will be species specific and differ according to age classes.

## Material and Methods

### Field work and logger deployment

The main study was carried out on Europa Island (22.3°S, 40.3°E), located in the Mozambique Channel, 300 km from the coast of Madagascar and 500 km from the mainland coast of Africa. The data were collected during the course of an extensive tracking programme focused on the foraging behaviour of the two species and carried out in 2014 between 27 January and 4 March^26,44^. During the study period, cyclone Guito (category 2) crossed the foraging grounds of the species in the Mozambique Chanel and hit Europa on 19–20 February 2014. Additional birds were fitted with loggers before the arrival of the cyclone.

For red-footed boobies, adults feeding chicks and juvenile boobies starting to fly were fitted with 20 g (32 × 22 mm) IGotU GPS loggers (Mobile Action Technology) to record their position every 2 min and 1 min, respectively. The GPS loggers were attached under three central tail feathers using Tesa tape^45^. Birds were chosen randomly and captured by hand or with a 6 m telescopic fishing pole fitted with a nylon noose for the birds nesting higher in the trees. A total of 380 tracks were collected from 7 adults and 34 juveniles, including 3 cases in which a juvenile and one of its parents were both tracked^44^.

For frigatebirds, adults feeding large chicks and juvenile birds were captured on or near the nests using a long telescopic pole equipped with a noose by day or by hand using night vision googles at night. Thirty-seven solar-charged GPS accelerometers (GPS/ACC, e-obs GmbH, Munich, Germany, recording altitude) were fitted to 19 breeding adult and 18 juvenile great frigatebirds to record flying. The devices measured 130 × 30 × 12 mm and weighed 30 g. They were attached to the back feather with waterproof tape (Tesa, Germany). The weight of the transmitters was 1.88–3.55% of the frigatebirds’ weight and 1.9–2.5% the weight of the boobies. These devices were recovered after one or several foraging trips.

In New Caledonia, 7 great frigatebirds from Walpole Island were fitted with 9.5 g solar-panelled Argos PTTs (duty cycle 12 hON, 12h OFF) in September 2014 and March 2015^46^. When cyclone Pam (category 4) crossed the New Caledonian area on 13–14 March, 4 non-breeding birds were present and foraging from islands around New Caledonia and Vanuatu.

### Data processing and wind and cyclone data

The Argos and GPS data were filtered using a speed filter, and speeds between two locations greater than 90 km.h^−1^ were excluded. Average speeds between two locations were calculated. We obtained data on wind speed and direction at the sea surface and various altitudes from https://earth.nullschool.net. These data are provided every 3 hours from GFS-NCEP models available from the US National Centre for Environmental Prediction (spatial resolution 25 km). For each location and altitude of the birds recorded using GPS or location for the Argos data, the wind data for the closest location and time were extracted. We then calculated the angle between the flight track of the bird between each location and the wind direction, with head winds at an angle of 180°, tail winds at 0°, and side winds at 90°.

At each colony, we used the wind data provided by an automatic station providing wind speed and direction data at 5 m above sea level every two hours (http://www.meteofrance.re). Here, we considered cyclonic conditions to occur when the average wind strength was higher than an average wind speed of 60 km.h^−1^, which correspond to winds associated with a cyclone or an intense tropical depression (Supplementary Materials, Fig. 1). We used the cyclone classification system from http://www.bom.gov.au/cyclone/about/intensity.shtml. Guito was classified as a category 2 cyclone when it hit Europa, with an average wind speed of 115 km/h and gust winds attaining 158 km/h. Cyclone Pam was a category 5 cyclone, with maximum average winds of 150 km.h^−1^and gust winds of 250 km.h^−1^.

### Statistical analyses

All values are given as the mean ± one S. D. unless stated otherwise. Because the individuals were tracked for several successive foraging trips from the colony where they were equipped, we analysed foraging parameters using mixed-model ANOVAs to account for pseudoreplication. Foraging parameters (trip duration, range, speed, altitude) were considered as dependent variables, sex was modelled as a fixed factor, and bird individual was included as a random factor. Since no sex effects were found, they were considered in the results. The statistical analyses were conducted using Statistica 13.

### Ethical statement

All methods, field procedures and animal handling were performed in accordance with the relevant guidelines and regulations. For Europa, the field procedures and manipulations were approved by the Préfet des Terres Australes et Antarctiques Françaises and Comité National de la Protection de la Nature. For New Caledonia, the field procedures and animal handling were approved by the Government of New Caledonia.

## Supplementary information

Supplementary material

## Data Availability

The data will be made available in Dryad and Movebank for tracking data.
