# Supplementary material for: Cyclone avoidance behaviour by foraging seabirds
Source: Sci Rep. 2019 Apr 1;9:5400. doi: 10.1038/s41598-019-41481-x (PMC6443659; doi:10.1038/s41598-019-41481-x)
Supplement: Supplementary file 1 — Supplementary material [file 41598_2019_41481_MOESM1_ESM.doc]

Electronic supplementary material

*Scientific Reports*

Cyclone avoidance behaviour by foraging seabirds

**Henri Weimerskirch**^1^ **& Aurélien Prudor**^1^

^1^ Centre d’Etudes Biologiques de Chizé, CNRS, 79360 Villiers en Bois, France


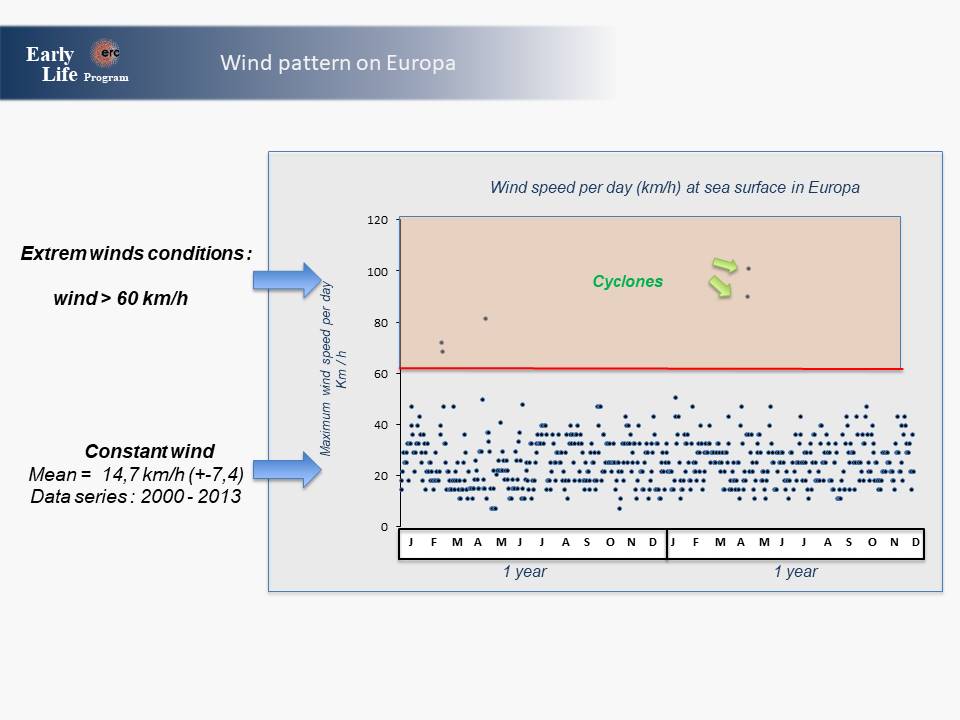


**Fig S1 Maximum winds recorded on Europa during two successive years, 2013 and 2014**
